# Supplementary material for: Ln(III) Complexes Embedded in Biocompatible PLGA Nanoparticles as Potential Vis-to-NIR Optical Probes
Source: Molecules. 2023 Feb 28;28(5):2251. doi: 10.3390/molecules28052251 (PMC10005321; doi:10.3390/molecules28052251)
Supplement: Supplementary file 1 [file molecules-28-02251-s001.zip › molecules-2222215-supplementary.pdf]

Ln(III) complexes embedded in biocompatible PLGA nanoparticles as potential Vis-to-NIR optical probes

Fabio Piccinelli [1], Silvia Mizzoni [1], Giorgia Zanella [2], Salvatore Calogero Gaglio [2], Massimiliano Perduca [2], Alessandro Romeo [3], Silvia Ruggieri [1,*], Chiara Nardon [1] and Enrico Cavalli [4]

1 Luminescent Materials Laboratory, DB, University of Verona, and INSTM, UdR Verona, Strada Le Grazie 15, 37134, Verona, Italy

2 Biocrystallography and Nanostructure Laboratory, Department of Biotechnology, University of Verona, Strada Le Grazie 15, 37134, Verona, Italy

3 Department of Computer Science, University of Verona, Strada Le Grazie 15, 37134, Verona, Italy

4 Department of Chemical Sciences, Life and Environmental Sustainability, University of Parma, Parco Area delle Scienze, 11/a, 43124, Parma, Italy

Supporting Information

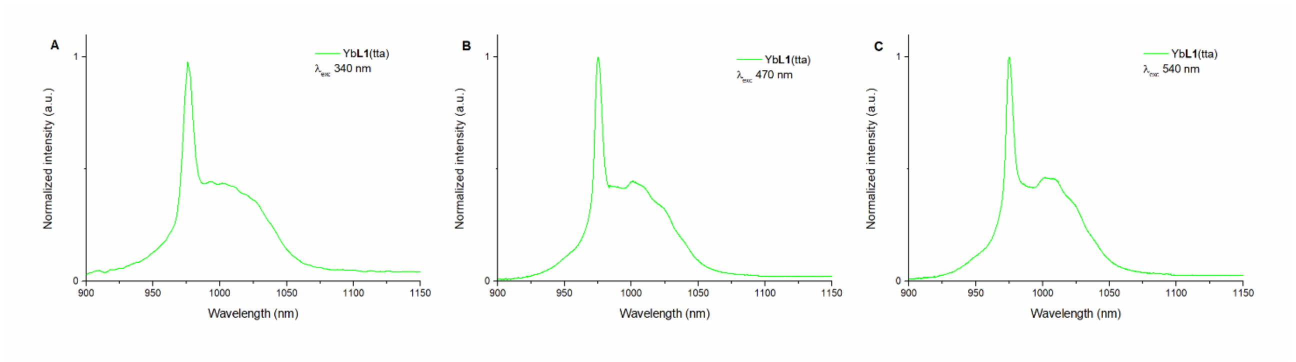

Figure S1. Luminescence emission spectra of (*R*,*R*)-Yb**L1**(tta) in methanol solution collected by exciting at 340 nm (A), 470 nm (B) and 540 nm (C). The spectra of (*S*,*S*) enantiomer (not reported) are superimposable.

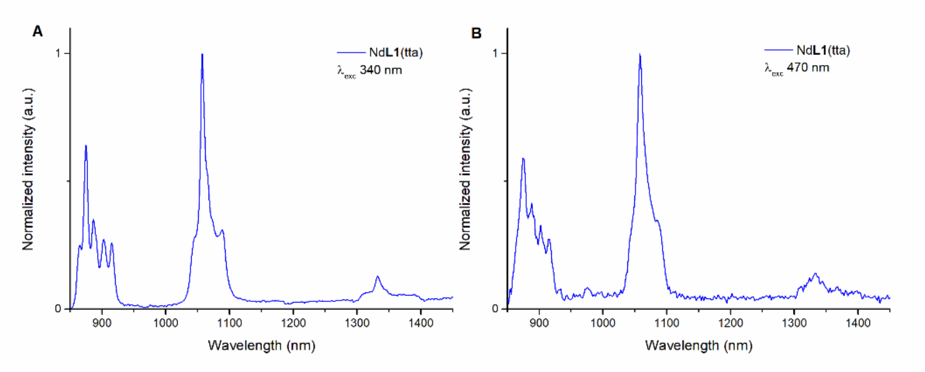

Figure S2. Luminescence emission spectra of (*R*,*R*)-Nd**L1**(tta) in methanol solution collected by exciting at 340 nm (A) and 470 nm (B). The spectra of (*S*,*S*) enantiomer (not reported) are superimposable.

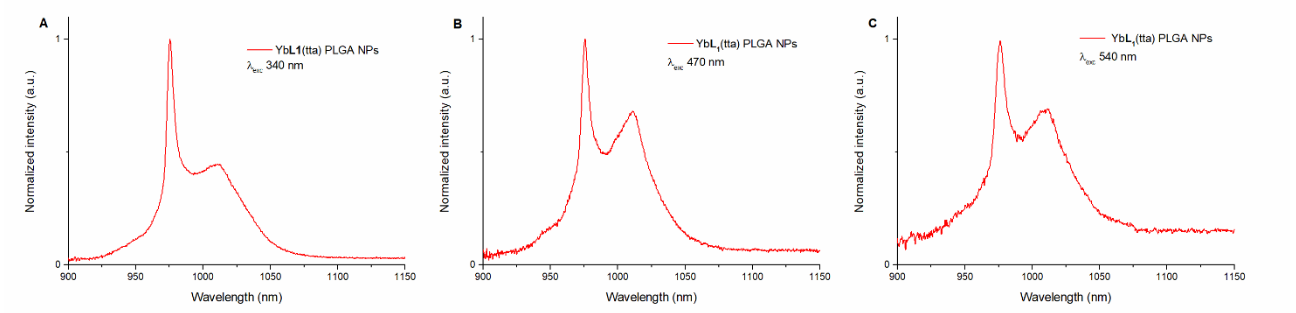

Figure S3. Luminescence emission spectra of (*R*,*R*)-Yb**L1**(tta) embedded in PLGA nanoparticles in water solution collected by exciting at 340 nm (A), 470 nm (B) and 540 nm (C). The spectra of (*S*,*S*) enantiomer (not reported) are superimposable.

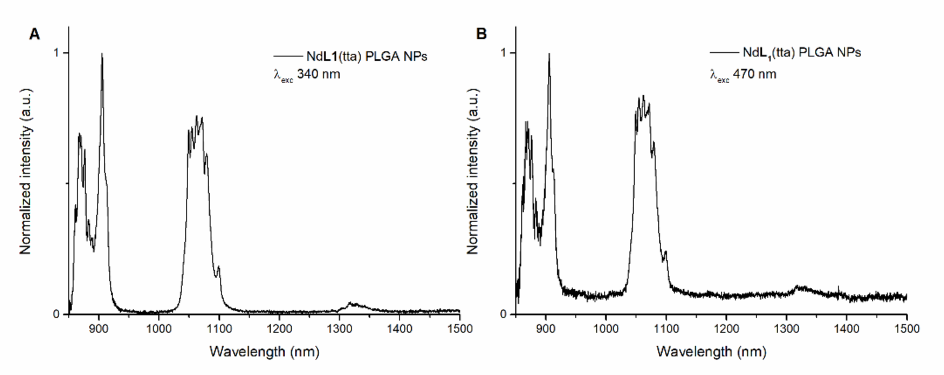

Figure S4. Luminescence emission spectra of (*R*,*R*)-Nd**L1**(tta) embedded in PLGA nanoparticles in water solution collected by exciting at 340 nm (A), 470 nm (B). The spectra of (*S*,*S*) enantiomer (not reported) are superimposable.

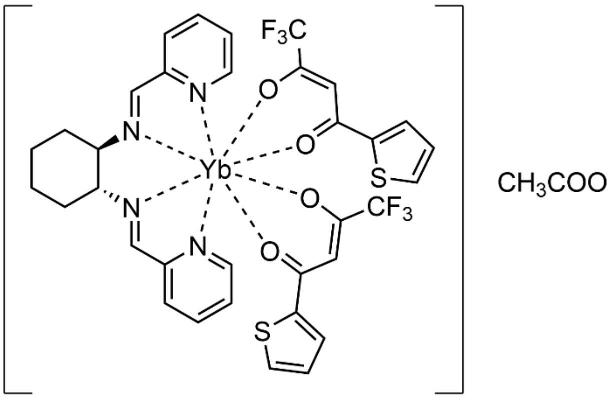

Figure S5. Molecular structure of [Yb**L**(tta)$_2$]CH_3COO investigated in a previous contribution. The (*R*,*R*) enantiomer, chosen as representative, is here reported.

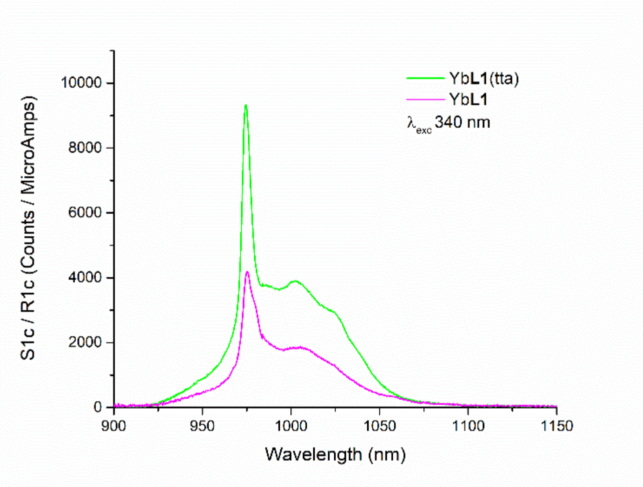

Figure S6. Overlap of the luminescence emission spectra in (50 μM) methanol solution related to (*R*,*R*)-Yb**L1**(tta) and (*R*,*R*)-Yb**L1**, upon excitation at 340 nm. The spectra have been collected using the same bandwidth, both in excitation and emission.

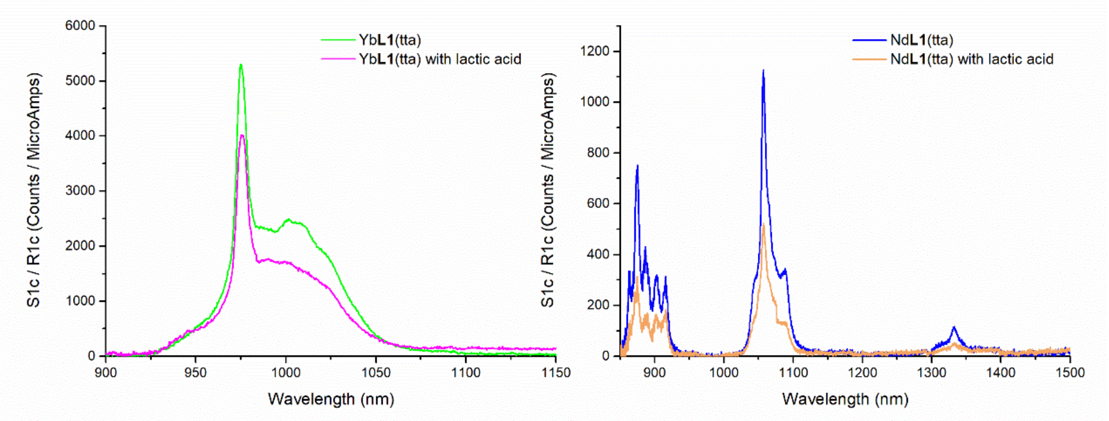

Figure S7. Evolution of the emission spectra at 20 °C, upon excitation at 470 nm, of (left) (*R*,*R*)-Yb**L1**(tta) and (right) (*R*,*R*)-Nd**L1**(tta), after 30 min from the addition of an excess of lactic acid.

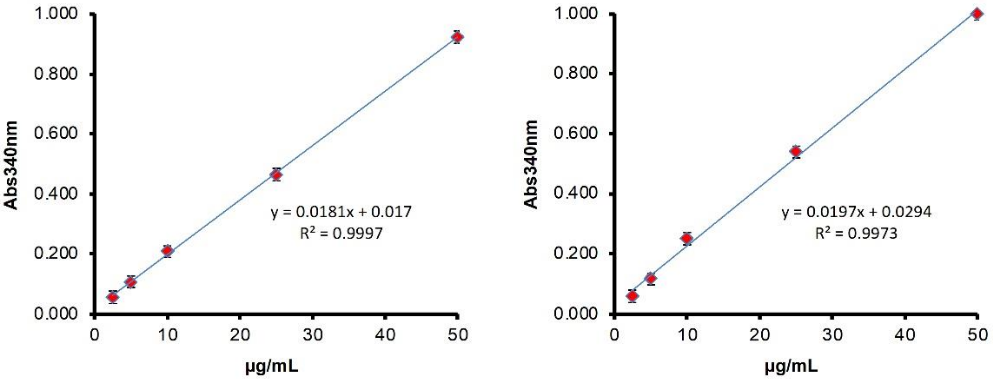

Figure S8. Calibration curves in acetonitrile [Absorbance at 340 nm of the complex vs conc (µg/mL)] of Yb (left) (EE =44.1% ± 4.4) and Nd (right) (EE = 63.1% ± 2.1).
